# The Dichotomic Role of Macrophage Migration Inhibitory Factor in Neurodegeneration

**DOI:** 10.3390/ijms21083023

**Published:** 2020-04-24

**Authors:** Maria Sofia Basile, Giuseppe Battaglia, Valeria Bruno, Katia Mangano, Paolo Fagone, Maria Cristina Petralia, Ferdinando Nicoletti, Eugenio Cavalli

**Affiliations:** 1 Department of Biomedical and Biotechnological Sciences, University of Catania, Via S. Sofia 89, 95123 Catania, Italy; sofiabasile@hotmail.it (M.S.B.); kmangano@unict.it (K.M.); paolofagone@yahoo.it (P.F.); eugeniocavalli9@hotmail.it (E.C.); 2 Department of Physiology and Pharmacology, Sapienza University, Piazzale A. Moro, 5, 00185 Rome, Italy; giuseppe.battaglia@uniroma1.it (G.B.); valeria.bruno@uniroma1.it (V.B.); 3 IRCCS Neuromed, Località Camerelle, 86077 Pozzilli, Italy; 4 Department of Educational Sciences, University of Catania, 95124 Catania, Italy; m.cristinapetralia@gmail.com

**Keywords:** macrophage migration inhibitory factor, neurodegeneration, amyotrophic lateral sclerosis, Parkinson disease, Huntington disease

## Abstract

Macrophage migration inhibitory factor (MIF) is a pleiotropic cytokine expressed by different cell types and exerting multiple biological functions. It has been shown that MIF may be involved in several disorders, including neurodegenerative disorders such as amyotrophic lateral sclerosis (ALS), Parkinson disease (PD), and Huntington disease (HD), that represent an unmet medical need. Therefore, further studies are needed to identify novel pathogenetic mechanisms that may translate into tailored therapeutic approaches so to improve patients’ survival and quality of life. Here, we reviewed the preclinical and clinical studies investigating the role of MIF in ALS, PD, and HD. The emerging results suggest that MIF might play a dichotomic role in these disorders, exerting a protective action in ALS, a pathogenetic action in HD, and a yet undefined and debated role in PD. The better understanding of the role of MIF in these diseases could allow its use as a novel diagnostic and therapeutic tool for the monitoring and treatment of the patients and for eventual biomarker-driven therapeutic approaches.

## 1. Macrophage Migration Inhibitory Factor (MIF)

MIF is a pro-inflammatory cytokine that was first discovered in 1966 as a product expressed by activated T lymphocytes and capable of inhibiting the migration of macrophages in vitro [1]. MIF is detectable in several cell types, such as epithelial, endothelial, and immune cells [2], as well as in various tissues of the endocrine system, in particular in the organs responsible for the response to stress, including the hypothalamus, pituitary glands, and adrenal glands [3]. Unlike other cytokines, MIF is stored in intracellular pools and thus it is promptly secreted [3]. The release of MIF into the extracellular environment occurs through a co-export process mediated by general vesicular transport factor p115 that is activated in response to stress stimuli such as Toll-like Receptor (TLR) ligands, mitogens, and pro-inflammatory cytokines [4]. MIF plays a key role in promoting the body’s response to inflammatory stimuli [5], promoting the secretion of mediators involved in the inflammatory response, including TNF-α, IL-1, IL-6, IL-8, IL-12, interferon (IFN)-γ, cyclooxygenase-2 (COX2), nitric oxide (NO), and matrix metalloproteinases (MMP) [5]. It has also been observed that MIF promotes the survival of macrophages by inhibiting p53e [5]. However, like for several other cytokines, pleiotropic effects have also been described for MIF that, under certain circumstances, may promote type 2 anti-inflammatory responses that seem to be primarily mediated by its ability to activate the AMPK pathway [3,5,6].

MIF also acts as an endocrine molecule, chaperone, and enzyme [5]. The endocrinological action of MIF is regulated by its secretion from the anterior pituitary and the adrenal gland in conjunction with activation of the hypothalamic-pituitary-adrenal (HPA) axis [5]. MIF also functions as a chaperon-like cytosolic protein and exhibits enzymatic properties characterized by D-dopachrome, phenylpyruvate tautomerase, Q3 activity, and also as thiol-oxidoreductase protein [5]. The activation of the MIF signaling pathway originates at the extracellular level following the link with the CD74 differentiation protein cluster and recruitment of the cell-surface glycoprotein CD44 signal transducer or interacting intracellularly with the JAB1/CSN5 receptor [3]. In addition, MIF acts as a ligand for the chemokine receptors, CXCR2, CXCR4, and CXCR7 [6]. In turn, MIF receptors interact with each other and establish four different units composed of two or more receptors to transduce the signaling pathway, such as CD74/CD44, CD74/CXCR2, CD74/CXCR4, and CD74/CXCR4/CXCR7 [7]. The interaction of MIF with the CD74-CD44 complex leads to the activation of the Src kinase, with consequent phosphorylation of the ½ kinase (ERK1/2) and mitogen-activated protein kinase (MAPK/ERK) [6]. Another interaction pathway of MIF and CD74 promotes the activation of the AKT pathway through the mediation of SRC and PI3K kinases and nuclear factor (NF)-kB pathways [3,6]. AKT activation determines the phosphorylation and inactivation of the pro-apoptotic proteins BAD and BAX, with inhibition of apoptosis. By activating AKT, MIF increases the expression of NF-kB and of the anti-apoptotic proteins Bcl-xL and Bcl-2 in lymphoid cells [7]. Furthermore, MIF stimulates the expression of cytoplasmic phospholipase A2 (cPLA2) [3] that favors the consequent expression of arachidonate and the subsequent release of prostaglandins E2 [3]. The production of intracellular arachidonate favors the post-transcriptional stability of the mRNAs that code for inflammatory cytokines following the activation of the N-terminal JUN kinase/stress-activated protein kinase (pJNK), which is inhibited by glucocorticoids [3]. MIF also interacts intracellularly with specific proteins [8] including, for example, the association of the JUN activation domain binding protein 1 (JAB1) with the signaling subunit COP9 5 (CSN5), with consequent inhibition of JAB activity induced by JAB1 and transcriptional AP-1 [8]. The interaction of MIF with Jab1/CSN5 leads to the activation of HIF1α and to the expression of pro-angiogenic factors IL-8 and VEGF [8]. The binding of MIF with JAB1/CSN5 also modulates AP-1 activity and cell proliferation by inactivation of p53 [8].

The d-dopachrome tautomerase (DDT, also known as. MIF-2), the second member of the MIF family, was discovered in 1997 [9]. DDT has a 34% homology with MIF and both share a common homotrimeric structure [9]. MIF and DDT showed common biological characteristics, such as the enzymatic activity represented by a catalytic proline residue [10]. As previously described for MIF, DDT also interacts with the CD74 receptor [10]. A fundamental difference consists in the absence of a binding domain that does not allow interaction with CXCR2 [10]. DDT is, however, able to activate the cascade of the MAP kinase ERK1/2, by activating the CD74/CD44 complex [10]. In a manner similar to MIF, DDT also binds intracellular JAB1/CSN5 receptors [10].

## 2. The Role of MIF in the Central Nervous System (CNS)

Several studies have investigated the expression of MIF and its possible role in the CNS. MIF protein has been found in ependymal cells, astrocytes, and neurons of the bovine brain and in the epithelial cells of the choroid plexus, ependymal cells, and astrocytes of the rat brain, whereas MIF mRNA has been identified both in astrocytes and neurons of the rat brain [11,12]. Moreover, a high baseline MIF expression has been found in neurons of the cortex, hippocampus, hypothalamus, cerebellum, and pons of rat brains [13]. Interestingly, MIF may play a key role in the proliferation of adult rodent hippocampal cells [14]. As regards the human brain, MIF is highly and widely expressed in neural tissues [15]. Furthermore, there are no significant variations in the levels of MIF in the CNS in the course of life, thus supporting the concept that MIF could play a homeostatic role due to its involvement in the isomerization of catecholamine derivatives to neuromelanin precursors [16]. Moreover, MIF seems to be involved in various inflammatory, vascular, traumatic, neoplastic, and neurodegenerative diseases of the nervous system, such as multiple sclerosis (MS), stroke and cerebral ischemia, autism-spectrum disorders, spinal cord injury, depression, glioblastoma, and Alzheimer disease (AD) [16,17,18,19,20]. Overall, the role of MIF in the CNS is not yet fully understood and further studies are warranted to clarify its precise mode of action in these conditions.

## 3. MIF in Neurodegenerative Diseases

Initial studies by ourselves and others have primarily evaluated the role of MIF in the pathogenesis of immunoinflammatory and autoimmune diseases and cancer [7,21,22,23,24,25,26].

However, because of its pleiotropic biological functions, during the last decades, many studies have investigated the involvement of MIF in neurodegenerative diseases [27]. The emerging results suggest that MIF may play both a protective or pathogenetic role in neurodegenerative disorders, in particular with a dichotomic role in AD and Parkinson disease (PD), a potential protective action in amyotrophic lateral sclerosis (ALS), and a possible pathogenetic role in Huntington disease (HD).

Since we have recently reviewed the role of MIF in AD [20], here we focused our attention on the involvement of MIF in ALS, PD, and HD.

## 4. ALS

ALS is a progressive neurodegenerative disease characterized by the death of alpha motor neurons provoked by heterogeneous pathogenetic pathways that involve the oxidative stress, the neuronal inflammation with immune cells infiltrating the CNS, the mitochondrial dysfunction, RNA splicing errors, and errors in protein conformation [27,28]. According to a study conducted in 2016, the incidence of ALS is approximately 1–2.6 cases per 100,000 people per year and that the prevalence is approximately 6 cases per 100,000 [29]. The correlation with the sex is calculated at 1:350 for men and 1:500 for women [30]. It has also been calculated that the average age for the onset of ALS is currently 58–60 years and that life expectancy is around 3–4 years after the onset of the disease [29]. ALS can be subdivided into sporadic ALS that occurs in 90–95% of cases and familial ALS that takes account for the remaining 5–10%, is of genetic origin, and it is also defined hereditary ALS [29].

Different studies have reported that the main cause of the onset of ALS is due to mutations in Cu/Zn superoxide dismutase gene (*SOD1*) [31], mutations in the *FUS* (fused in sarcoma) gene, which encodes a protein responsible for DNA repair and related to juvenile-onset forms of the disease or *TDP-43* (TAR DNA-binding protein 43), a key protein for repair pathway of DNA double-strand breaks in motor neurons and oligodendrocytes [32,33]. The most common hereditary cause of ALS is the expansion of hexanucleotide repeat (GGGGCC) in the noncoding region of the *C9ORF72* gene, which leads to loss of protein transcription [34,35]. Even though mutations in all the mentioned genes are more frequent in familial form of ALS, they are present also in sporadic cases [32,33,34,35]. As previously mentioned, ALS is a disease characterized by the loss of motor neurons in the CNS [36] that provokes the inability to control voluntary movements and consequently respiratory failure and difficulty in swallowing occur [36]. Of all the causes listed above, the different gene mutations affecting the superoxide dismutase gene are currently the most studied [31,36].

There are no effective therapies for ALS with the only two drugs approved for the disease being riluzole (Riluteck^®^, Sanofi-Aventis) and edaravone (Radicut^®^, Mitsubishi Tanabe Pharma), that only slow the course of the disease by a few months. Riluzole works by reducing excitotoxicity while edaravone reduces oxidative stress [37].

## 5. MIF in ALS

The emerging results from preclinical in vitro and in vivo studies investigating the role of MIF in ALS suggest that MIF may exert potential protective effects in ALS [27]. The pathogenesis of ALS is still unknown, but as previously indicated, mutant SOD1 could play a key role in this pathology [31] through the mitochondrial accumulation of mutated SOD1 that causes mitochondrial dysfunction and subsequent death of motor neurons [38]. Mutant SOD1 could act by accumulating within the intermembrane space (IMS) thus bypassing the physiological retention regulated by the copper chaperone for superoxide dismutase (CCS) or by deposition on the external mitochondrial membrane (OMM) with blockade of the transport through the mitochondrial membranes [38].

Several in vitro and in vivo studies have shown that MIF can inhibit the accumulation of misfolded SOD1 [36,39]. MIF can regulate both intracellular and extracellular pathways. Intracellularly, MIF acts as a chaperone protein and a thiol-oxidoreductase protein [36]. Its protein folding activity derives from the transition from multimeric to monomeric forms, thus exposing a hydrophobic surface that can provide chaperone activity ATP independent [38,40]. SOD1 has been observed to be normally localized both in the cytoplasm and in the cell nucleus. MIF chaperone activity may inhibit SOD1 misfolding [36,38,40]. At the nuclear level, it has been observed that the misfolded SOD1 generates a sequence similar to a nuclear export signal (NES), which is normally inactive in normal SOD1, allowing the removal of misfolded SOD1 from the nucleus to the cytosol by the protein of nuclear transport CRM1 [36].

The inhibition of misfolded SOD1 nuclear export by MIF is due to its chaperone activity in the nucleus, preventing the exposure of the NES sequence with subsequent release and accumulation of misfolded SOD1 in the cytosol [36]. At the cytosol level, MIF catalytically inhibits the accumulation of SOD1 and its association with mitochondria and ER [36,40].

In particular, SOD1 interactions with mitochondria and OMM proteins, such as Bcl-2 and VDAC, lead to activation of the pro-apoptotic mitochondrial pathway [38,40]. MIF chaperone activity prevents the binding of SOD1 with OMM proteins and inhibits the pro-apoptotic cell pathway and the accumulation of SOD1 misfolded in the cytosol [38]. In particular, the ability of MIF to suppress the toxicity of SOD1 misfolded in motor neuron-like cells may be due to changes in the aggregation model from amyloid aggregates to amorphous aggregates [36]. In particular, in in vitro studies, MIF chaperone activity inhibits the formation and toxicity of misfolded SOD1 amyloid aggregates, when overexpressed in neuroblastoma cell lines such as SH-SY5Y or mouse motor neuron-like hybrid cell line NSC-34 differentiable in motor neurons [36,39]. Studies in animal models of ALS have validated the potential beneficial effects of endogenous MIF [39,41]. Mice with mutant *SOD1* genes that were knocked down for *MIF* exhibited higher amounts of misfolded SOD1 in the spinal cord, along with an accelerated onset of the disease and reduced the lifespan as compared to the *MIF* wild-type control group [39,41].

**Figure 1:**
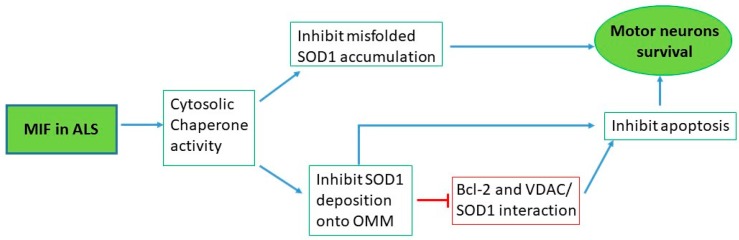
MIF in ALS. Preclinical studies on MIF in ALS which show the results obtained in vitro and in vivo and the correlation between MIF chaperone activity and its protective effect against mutant SOD1 toxicity in motor neurons. MIF also inhibits the activation of pro-apoptotic mitochondrial pathway, blocking misfolded SOD1 interactions with outer mitochondrial membrane proteins, such as Bcl-2 and VDAC [38].

A summary of the preclinical evidences pointing to a role for MIF in ALS is provided as Figure 1.

### 5.1. Preclinical Studies

#### 5.1.1. In Vitro Studies

A recent study reported that it is possible to reproduce in vitro ALS model, in the neuroblastoma cell line NSC-34 that can be differentiated in motor neurons after treatment with retinoic acid [42].

A study aimed at evaluating whether MIF might act as a chaperone and can inhibit the accumulation of misfolded SOD1 [40]. The study reports that recombinant MIF inhibits the association of mutant SOD1 with mitochondria in a dose-dependent manner and increased accumulation of MIF enhances survival of primary motor neurons expressing mutant SOD1 [40]. Besides, augmented levels of MIF in neuronal cells suppresses the accumulation of misfolded SOD1 and its association with mitochondria and the ER extends survival of mutant SOD1-expressing motor neurons [40].

Another study showed that MIF inhibits mutant SOD1 nuclear aggregation when overexpressed in motor neuron-like NSC-34 cells [36]. Also, MIF altered the typical SOD1 amyloid aggregation pathway in vitro and, instead, promoted the formation of disordered aggregates, as measured by Thioflavin T (ThT) assay and as also shown by transmission electron microscopy (TEM) imaging [36]. Moreover, MIF reduced the toxicity of misfolded SOD1 by directly interacting with it. Vice versa, the chaperone function and protective effect of MIF in neuronal cultures do not require its intrinsic catalytic activities [36]. Altogether, this study implicates MIF as a potential therapeutic candidate in the treatment of ALS [36].

Finally, another study was performed to test in vitro if increased synthesis of MIF can prevent the accumulation of misfolded SOD1 and protect against its toxicity in the SH-SY5Y neuroblastoma cell line [39]. Neuron cell lines were transfected to express the human WT (*SOD1WT*) or mutant (*SOD1G93A*) *SOD1* transgenes with or without co-transfection with a plasmid encoding for the human *MIF* [39]. This latter manipulation resulted in an increase of MIF expression in neuronal cultures that inhibited the accumulation of misfolded SOD1 and rescued from mutant SOD1-induced cell death [39].

#### 5.1.2. In Vivo Studies

The potential beneficial role of MIF in ALS that was proposed by the in vitro studies was further strengthened by in vivo studies showing that endogenous MIF acts as a chaperone for misfolded SOD1 [39]. Leyton-Jaimes showed that eliminating MIF in a mutant SOD1 mouse model of familial ALS increased the buildup of misfolded SOD1 [39]. They bred *MIF*-deficient mice with *SOD1G85R* mice, which express a dismutase-inactive mutant of SOD1. It was demonstrated that accumulation of misfolded SOD1 was localized in mitochondrial and ER membranes, and also resulted in higher levels of insoluble SOD1 aggregates in the spinal cords of *SOD1G85R-MIF*(-/-) mice than in their *SOD1G85R-MIF*(+/+) [39]. *MIF* KO mice expressing mutant SOD1 showed an early disease onset with a reduction of the lifespan [39]. The same group evaluated in a second in vivo study whether MIF overexpression was capable of inhibiting SOD1 mutant misfolding [41]. For this purpose, the mutant *SOD1G93A* and *loxSOD1G37R* mice were injected with adeno-associated viral vectors (AAV) to overexpress MIF [41]. The treatment led to overexpression of mRNA and MIF protein levels in the spinal cords of mice injected with AAV2/9-MIF [41]. It was demonstrated that the mutant *SOD1G93A* and *loxSOD1G37R* mice injected with *AAV2/9-MIF* exhibited a significant delay in disease onset and prolonged survival compared to non-injected *AAV2/9-GFP* controls [41]. Furthermore, the mice treated with *AAV2/9-MIF* had low amounts of SOD1 poorly folded in their spinal cords, without showing the hyperactivation of the glia following the upregulation of MIF [41]. The results of the study further indicate that MIF plays a fundamental role in the processing of SOD1 [41].

### 5.2. Clinical Studies

To the best of our knowledge no clinical studies have studied circulating levels and/or in vitro production of MIF or its homologue DDT in ALS patients. These studies are clearly warranted for the better understanding of the role of MIF in the pathogenesis of ALS.

## 6. PD

PD was first identified in 1817 by James Parkinson, who wrote the “Essay on the shaking palsy” reporting a disease characterized by motor signs such as bradykinesia, rigidity, and tremor, and mental symptoms [43]. With a prevalence of 1–2 per 1000 in unselected populations and of 1% in the population above 60 years, PD represents the most frequent movement disorder and the second most frequent neurodegenerative disease of the CNS [43]. While less than 10% of PD cases are familial, the majority of PD cases are sporadic and may depend on a combination of multiple factors, including age, gender, genetic, and environmental factors [44]. Male sex and increasing age may be independent risk factors, whereas smoking and coffee drinking seem to be protective factors [45]. About 30% of the familial and 3–5% of the sporadic PD cases are monogenic forms that derive from a single mutation in a gene and that can be inherited dominantly, such as *SNCA (PARK1 = 4),* and *LRRK2 (PARK8*) or recessively, such as *Parkin (PARK2), PINK1 (PARK6), DJ-1 (PARK7),* and *ATP13A2 (PARK9)* [46]. Moreover, it seems that all familial PD cases are correlated with mutations in genes directly or indirectly involved in mitochondrial dysfunction [44].

PD is characterized by the presence of Lewy bodies that are primarily made by α-synuclein, and by the loss of dopaminergic neurons in the substantia nigra [47]. The most common symptoms of PD are bradykinesia, tremor, rigidity, and postural instability [48]. Unfortunately, when these signs appear, there is a loss of more than half of nigrostriatal dopaminergic terminals [48]. Hence, the discovery of diagnostic biomarkers that may select individuals at risk to develop PD would be of great relevance [48]. There are different PD subtypes and, besides the characteristic motor symptoms, there are a large variety of other symptoms, including psychiatric and cognitive dysfunctions [48]. Moreover, nonmotor symptoms, such as dysautonomia, hyposmia, rapid eye movement, and sleep disorders, could anticipate motor symptoms [48].

Currently, the most effective treatment for PD remains the first identified treatment that is levodopa and that is usually administered along with carbidopa [45]. Levodopa is the precursor to dopamine (DA) and can pass the blood-brain barrier, thus contributing to a major release of DA from the reduced number of dopaminergic neurons [45]. Moreover, DA agonists, such as pramipexole, ropinirole, and rotigotine, are used to stimulate dopaminergic receptors in the CNS, thus improving PD symptoms [45]. Monoamine oxidase aldehyde dehydrogenase B (MAO-B) inhibitors, e.g., rasagiline and selegiline, and catechol-O-methyl transferase inhibitors, such as entacapone, are used in PD treatment with the aim to extend the effectiveness of carbidopa/levodopa [45]. Furthermore, anticholinergic medications are used to reduce rigidity, dystonia, and tremor and tricyclic antidepressants, serotonin-noradrenaline reuptake inhibitors, and serotonin reuptake inhibitors may be administered in case of depression that is a common comorbidity associated with PD [45]. In cases where primary treatment is not effective, deep brain stimulation may be a potential therapeutic strategy [45]. Another interesting possible approach to treat PD is represented by nigral cell transplantation [44]. Moreover, a promising treatment option seems to rely on gene therapy aimed at correcting PD-related genes or at increasing neurotrophic factors, by using CRISPR technology, viral vectors, small interfering RNA (siRNA), or microRNA (miRNA) [44]. Promising results in reducing the risks of PD development have been observed with natural products such as green tea, coffee, and cotinine, a nicotine metabolite [44]. Considering the multifactorial etiology and the different subtypes of PD, tailored approaches of precision medicine are needed [44].

The current lack of fully effective preventive and curative treatments makes PD a clear unmet medical need and warrants studies aimed at identifying novel therapeutic approaches that either alone or in combination with existing treatments may improve the therapeutic outcome.

## 7. MIF in PD

**Figure 2:**
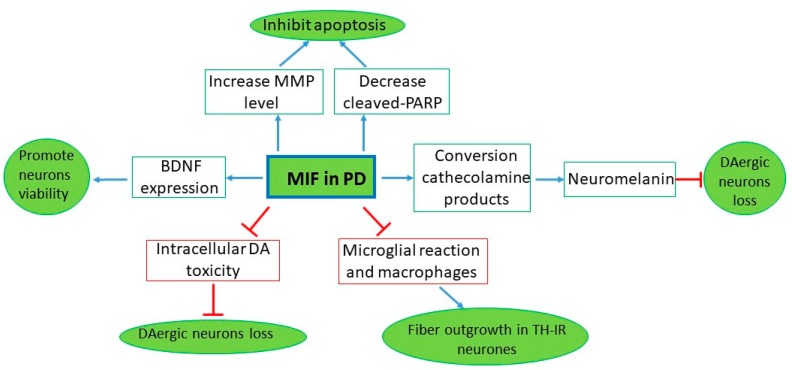
MIF in PD. Preclinical studies on MIF in PD, which show the results obtained in vitro and in vivo studies and the different pathway of how MIF could exert its protective function to inhibit neuronal loss. MIF mediates a neuroprotective effect via suppressing inflammatory responses, reducing the number of microglia and macrophages and displaying tyrosine hydroxylase-immunoreactive (TH-IR) neurons’ enhanced fiber outgrowth [49]. MIF also inhibits apoptosis and neuronal loss, detoxifying catecholamine-derived [15] and also promoting BDNF expression [50], increasing MMP and decreasing cleaved-PARP [47].

During the last decades, several preclinical and clinical studies have investigated the involvement of MIF in PD. The results generated are somehow conflicting and seem to suggest a possible dichotomic role of this cytokine in PD. Indeed, MIF might either contribute to disease progression by enhancing neuroinflammation or exert a protective action due to its involvement in catecholamine detoxification (Figure 2).

### 7.1. Preclinical Studies

#### 7.1.1. In Vitro Studies

Matsunaga et al. have shown that MIF is highly expressed in adult human brain tissues and can catalyze the conversion of two toxic catecholamines-derived quinone products to indoledihydroxy derivatives that may be neuromelanin precursors [15]. Hence, they suggested that MIF could be involved in the detoxification of catecholamine products and might, therefore, exert a protective action in PD neural tissues which are damaged by catecholamine-related cell death [15].

Considering the possible involvement of DA in PD and that of MIF in DA metabolism, Weingarten et al. investigated the role of MIF in protection against DA cytotoxicity in amino acid decarboxylase (AADC)-expressing Chinese hamster ovary (CHO) cells transfected with *MIF* [51]. The authors found that MIF exerted a protective action against intracellular, but not extracellular, DA cytotoxicity and also against l-DOPA toxicity [51]. Since MIF is highly expressed in the brain, including the substantia nigra, it could exhibit a protective effect for dopaminergic neurons in vivo [51]. Overall, they suggested that the progressive neurodegeneration in PD might be due to dysfunction in DA disposition and/or metabolism and that genetic or chemical therapeutic intervention to upregulate the above-mentioned protective mechanisms could be promising [51].

Nakahara et al. found that MIF was S-nitrosylated by a physiological NO donor in vitro and that MIF activity was reduced after S-nitrosylation [50]. Chronic nitrosative stress may be associated with neurodegeneration [50]. Moreover, MIF is involved in the control of inflammatory responses and also increases the expression of brain-derived neurotrophic factor (BDNF), which favors the viability of neurons [50]. Therefore, they suggested that MIF dysfunction caused by NO might be involved in the loss of neuronal viability associated with neurodegenerative diseases, such as PD [50].

Li et al. investigated the effects of MIF in an in vitro model of PD induced in the SH-SY5Y cell line by the mitochondrial toxin MPP+, that is an inhibitor of complex I of the electron transport chain [47]. *MIF* was upregulated in this cellular PD model by using lentivirus, and it was downregulated by siRNA and by treatment with the MIF inhibitor ISO-1 [47]. Early apoptotic cells were increased in PD-like cells [47]. Interestingly, PD cells with upregulated *MIF* showed a reduced concentration of cleaved-PARP than the control group, higher mitochondrial membrane potential (MMP) than the *MIF* knockdown group, increased concentration of LC3B-II than the control group and increased LC3 puncta [47]. Moreover, MIF was negatively correlated with TNF-α and positively correlated with IL-10 [47]. Overall, the authors found that MIF exerted a neuroprotective action in this model, increasing cell viability that was associated with suppression of inflammatory response, enhanced autophagy, and inhibited apoptosis via the increase of the MMP [47].

#### 7.1.2. In Vivo Studies

Schwarz et al. investigated the effects of MIF on the function and survival of allogeneic fetal mesencephalic dopaminergic grafts in the 6-hydroxydopamine rat model of PD [49]. Although the activated microglia is involved in intracerebral grafts’ rejection, the authors found that MIF treatment, administered via intracerebral injections, was able to decrease microglia and macrophages in the grafts without improving grafts’ function and survival [49].

Li et al. studied MIF also in vivo in acute and chronic models of PD, obtained through the injection of 1-methyl-4-phenyl-1,2 ,3,6-tetrahydropyridine hydrochloride (MPTP), in C57BL/6 mice [47]. The authors found that the expression of MIF, cleaved-PARP, and LC3B-II was increased significantly in both models and that MIF was positively associated with IL-10, but inversely with TNF-α. In addition, the PD cells induced in vitro by challenge of SH-SY5Y cells with 1 mM MPP showed an increase in early apoptotic cells by FACS that was reduced by upregulation of MIF expression. The MMP was higher in the MIF upregulated group than in the *MIF* knockdown PD cells. In addition, upregulated MIF expression led to a higher concentration of LC3B-II than the control group. Finally, LC3 puncta were markedly increased in the *MIF* upregulated group and in the MIF + MPP+ group. This study indicates that MIF mediates a neuroprotective effect via suppressing inflammatory responses, inhibiting apoptosis, and inducing autophagy in PD [47].

In contrast to the two previous in vivo studies, Cheng et al. demonstrated that microglial autophagy deficiency in mice increased neuroinflammation, determined PD-like symptoms, and enhanced mRNA and protein brain MIF levels in a NLR family pyrin domain containing 3 (NLRP3) inflammasome-dependent manner [52]. Moreover, they found that MCC950, a specific NLRP3 inhibitor, inhibited NLRP3 inflammasome activation and reduced MIF expression and neuroinflammation, thus rescuing neuronal loss in the substantia nigra [52].

### 7.2. Clinical Studies

We have first demonstrated that MIF blood levels were significantly increased in PD patients as compared to healthy controls and epileptic patients [53]. However, there were no significant differences in *MIF* mRNA expression in the peripheral blood cells between these groups, thus suggesting that the increase originated from neuronal tissues [53]. Moreover, there was no correlation between the increase of MIF levels and the severity of the disease. However, this could have depended on the high homogeneity of the patients enrolled and the pharmacological treatment. Subsequently, Cheng et al. confirmed that serum levels of MIF are increased in PD patients and also demonstrated a significant association with PD development. The reasons for the discrepancy, as regard the correlation between MIF levels and PD progression between our study [53] and that of Cheng et al. [52], remain to be clarified [53]. As discussed above, although the upregulation of MIF levels in PD patients could favor disease progression by increasing neuroinflammation, it might also be considered as a compensatory mechanism of catecholamine detoxification [53]. Additional studies are also needed to better understand whether or not MIF may represent a promising biomarker for PD progression and, eventually, therapeutic response and in particular so since the finding may not be disease-specific as increased serum levels of MIF have also been reported in other neurological disorders, including AD and MS [20,21,23,24].

## 8. HD

HD was first reported in 1872 by George Huntington, who described a progressive and hereditary chorea, correlated with psychiatric and cognitive symptoms, that appears in individuals between 30 and 40 years of age [54].

HD prevalence is highly variable between different populations and ethnic groups, probably depending on genetic differences in the *HTT* gene [54]. Indeed, Western populations show a prevalence of HD of 10.6–13.7 persons per 100,000, whereas Japan, Taiwan, and Hong Kong present a prevalence of 1–7 per million and white and mixed populations have a higher prevalence than black populations in South Africa [54].

HD is a progressive neurodegenerative disease with autosomal dominant inheritance that usually appears in early adulthood and presents motor, cognitive, and behavioral dysfunction [55]. It is caused by an expanded trinucleotide (CAG) repeat of different length in *HTT*, the gene located at chromosome 4p16.3 that encodes the protein huntingtin [55]. While the CAG repeat number in *HTT* in the general population may vary from 6 to 35, there is an incomplete HD penetrance in individuals with 36–39 CAG repeats and a complete penetrance in individuals with 40 and more CAG repeats [56]. Although it is known that huntingtin may be involved in nervous system development, in cell adhesion, and in BDNF production and transport, the normal function of huntingtin is still not fully understood [55]. The mutant form of the protein huntingtin presents abnormally long polyglutamine sequences that correspond to the CAG genetic repeat, give toxic properties, and may lead to protein fragmentation, thus causing neuronal impairment and death [55]. In particular, in addition to its direct toxicity, the mutant huntingtin may determine the dysfunction and death of neurons via different pathogenetic cellular mechanisms, including interruption of cellular proteostasis, transcription, and mitochondrial function [54]. At the macroscopic level, the disease may at first modify the striatum and subsequently the cortex [54].

HD diagnosis relies on motor symptoms, family history, or positive genetic testing [54]. The motor dysfunctions are identified according to the Unified HD Rating Scale (UHDRS) total motor score (TMS) diagnostic confidence score, that may vary from 0, when there are any motor disturbances indicating HD, to 4, when there is a probability ≥99% of HD [54]. However, in certain cases, a family history could be absent, and ≈1% of patients that present a clinical situation suggesting HD may result in negative to the genetic test [57]. It is possible that these patients have other genetic mutations named “HD phenocopies”, such as the *C9orf72* hexanucleotide repeat expansion mutation [57].

Since there are no disease-modifying treatments for HD, the clinical care of patients is based on multidisciplinary supportive and symptomatic management, that requires a combination of pharmacological and nonpharmacological treatments and the expertise of different healthcare professionals, including physicians, nurses, physiotherapists, speech and language therapists, dieticians, and others [54].

During the last years, several promising approaches for HD treatment have been investigated in preclinical and clinical studies [57]. Among these, there are *HTT* gene silencing and reduction of huntingtin expression, e.g., genome editing techniques, including zinc finger proteins and CRISPR/Cas9, and post-transcriptional inhibition, including RNA interference and antisense oligonucleotides [57]. Moreover, another interesting approach is represented by targeted small molecule therapeutics that may modify the mutated huntingtin at the post-translational level, e.g., by enhancing phosphorylation at specific neuroprotective residues [57]. Recently completed and ongoing clinical trials have investigated treatments to decrease the levels of mutant huntingtin (e.g., antisense oligonucleotide therapy), immune modulators, stem cell therapy, deep brain stimulation, cognitive therapy, and specific physical activity [58].

## 9. MIF in HD

**Figure 3:**
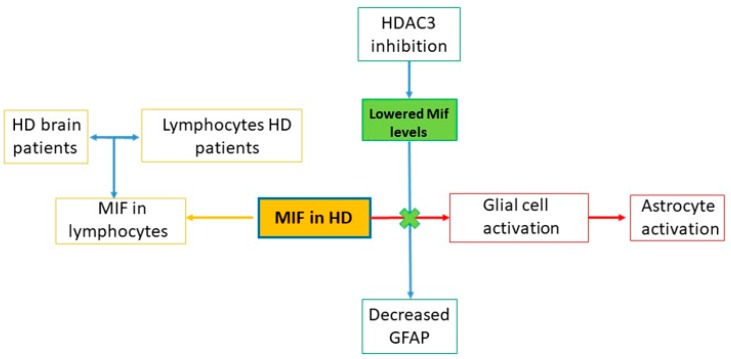
MIF in HD. Preclinical studies on MIF in HD show that lymphocytes of HD patients express MIF against a single agent present in brain samples of HD patients [59]. It has also been observed that MIF down expression, through the inhibition pathway of HDAC3, reduces the activation of the astrocytic response [60].

To date, the role of MIF in HD has been investigated only in a few preclinical studies, that suggest that MIF may be involved in HD pathogenesis (Figure 3). However, further studies are warranted in order to clarify the exact role of MIF in this neurodegenerative disorder.

### 9.1. Preclinical Studies

#### 9.1.1. In Vitro Studies

The possible involvement of MIF in HD was first reported by Barkley et al., who found that lymphocytes from HD patients, but not from healthy controls, produced MIF, when cultured with the extracts of HD brain tissue [61]. On the other hand, HD lymphocytes responded only rarely to the extracts of the brain tissue from the healthy controls [61]. Therefore, the authors suggested that the HD immune response mediated by MIF may be directed against an agent present only in the HD brain [61]. Subsequently, Barkley et al. discovered that lymphocytes from HD patients, but not those from MS patients, produced MIF in response to the presence of HD and MS brains [62]. Another study from the same group confirmed the production of MIF by lymphocytes from HD patients in response to antigen present in HD and MS brain tissues but not in healthy brain tissues [59].

#### 9.1.2. In Vivo Studies

Since histone deacetylase 3 (HDAC3) is involved in neuronal toxicity, in huntingtin-induced cell death, and immune events, Jia et al. investigated the effects of a selective HDAC3 inhibitor, named RGFP966 ((E)-N-(2-amino-4-fluorophenyl)-3-(1-cinnamyl-1H-pyrazol-4-yl)acrylamide) in a murine model of HD [60]. RGFP966 treatment improved motor deficits and exerted a neuroprotective action with significantly decreased striatal gene expression of *MIF* in the *N171-82Q* transgenic mouse model of HD but not in WT mice [60]. Moreover, RGFP966 reduced glial fibrillary acidic protein (GFAP) immunoreactivity, thus indicating a decreased astrocyte activation, in the striatum of *N171-82Q* mice compared to mice treated with vehicle [60]. Therefore, this study suggests that the HDAC3 inhibition could be a promising therapeutic strategy for HD patients and might be mediated by the reduction of *MIF* and the consequent possible downstream effects, including reduced astrocyte activation [60].

## 10. Conclusions

Many neurodegenerative diseases, including ALS, PD, and HD, still represent an unmet medical need and new therapeutic approaches are strongly warranted to improve both patients’ survival and quality of life. During the last decades, the study of the potential role of MIF in neurodegenerative diseases has attracted increasing interest because of its ability to modulate various functions both in physiological and pathological conditions of the CNS.

This review highlighted the involvement of MIF in neurodegenerative diseases, in particular in ALS, PD, and HD. The emerging results from the current preclinical and clinical studies suggest that MIF might play different roles in these neurodegenerative disorders. While MIF seems to exert a protective action in ALS, it might exert either a positive or negative action in PD and may be pathogenetically involved in HD development. In this review we highlighted current concepts on the physiological and physiopathological role of MIF in the regulation of CNS processes and functions and the possible influence it plays on these neurodegenerative diseases. The data reviewed strengthen the concept that MIF possesses biological properties that makes it capable of influencing, in different manners, the development of ALS, PD, and HD. This evidence propels the tailored combination of additional preclinical experiments along with clinical studies including measurement of circulating levels of MIF, its variation in response to treatment, and correlation with disease progression that may help achieve a more complete understanding of its role in these diseases with the ultimate aim to dismantle whether novel therapeutic approaches eventually based on MIF agonisms or antagonisms can be considered for these diseases.

In particular, if MIF plays a protective role, the possibility to administer tailored agonistic approaches, such as exogenously administered MIF or small molecule MIF agonists, should be considered. Conversely, if MIF exerts a pathogenetic action, specific MIF antagonists, such as anti-MIF monoclonal antibodies or small molecule inhibitors, could represent a valuable possibility of complementary therapy in certain cases and phases of these neurodegenerative diseases. Tailored MIF inhibitors that are already in clinical trials or already approved for other conditions deserve particular attention due to their possible faster translation to the clinical setting. Among these there are the anti-MIF mAb inhibitor Bax69 that is in Phase I/II clinical trials for cancer patients, the anti-CD74 mAb milatuzumab that is approved for hematological malignancies, and the tautomerase inhibitor Ibudilast that is clinically used in bronchial asthma treatment and is being repurposed for immunoinflammatory conditions [7,20].

Furthermore, the potential role of the second member of the MIF family, DDT, in ALS, PD, and HD is yet unexplored and studies on the possible synergisms of these two analogues in promoting pathways responsible for disease induction or protection are highly warranted.
